# Local anesthetic levobupivacaine inhibits stemness of osteosarcoma cells by epigenetically repressing MAFB though reducing KAT5 expression

**DOI:** 10.18632/aging.203975

**Published:** 2022-03-25

**Authors:** Zhan Wang, Yuxin Song, Hui Zhang, Yang Yang, Suifeng Zhang, Wenji Wang

**Affiliations:** 1 The First School of Clinical Medicine of Lanzhou University, Department of Orthopaedics, The First Hospital of Lanzhou University, Lanzhou 730000, Gansu, China; 2 Department of Orthopaedics, Gansu Provincial Hospital, Lanzhou 730000, Gansu, China

**Keywords:** osteosarcoma, CSCs, levobupivacaine, KAT5, MAFB

## Abstract

Osteosarcoma is the most prevalent bone cancer and accounts for over half of sarcomas. In this study, we identified that the treatment of levobupivacaine suppressed proliferation of osteosarcoma cells *in vitro*. The tumor xenograft analysis showed that levobupivacaine significantly repressed the osteosarcoma cell growth in the nude mice. The treatment of levobupivacaine improved the apoptosis rate and attenuated invasion and migration abilities of osteosarcoma cells. The sphere formation capabilities of osteosarcoma cells were repressed by levobupivacaine. The protein levels of Sox-2, Oct3/4, and Nanog were inhibited by the treatment of levobupivacaine in osteosarcoma cells. Regarding mechanism, we identified that levobupivacaine inhibited MAFB and KAT5 expression in osteosarcoma cells. We observed that lysine acetyltransferase 5 could enriched in the promoter region of MAF BZIP transcription factor B, while levobupivacaine treatment could repressed the enrichment. The suppression of KAT5 by siRNA repressed the enrichment of histone H3 acetylation at lysine 27 and RNA polymerase II on promoter of MAFB. The expression of MAFB was decreased by KAT5 knockdown in osteosarcoma cells. The expression of MAFB was repressed by levobupivacaine, while the overexpression of KAT5 could reverse the repression of MAFB. KAT5 contributes to the cell proliferation and stemness of osteosarcoma cells. The overexpression of KAT5 or MAFB could reverse levobupivacaine-attenuated cell proliferation and stemness of osteosarcoma cells. Therefore, we concluded that local anesthetic levobupivacaine inhibited stemness of osteosarcoma cells by epigenetically repressing MAFB though reducing KAT5 expression. Levobupivacaine may act as a potential therapeutic candidate for osteosarcoma by targeting cancer stem cells.

## INTRODUCTION

Osteosarcoma is the most frequently occurring bone cancer and accounts for over half of sarcomas [1]. Epidemiological studies indicated that children and adolescents are more susceptible to this cancer [1]. Although the surgical techniques and neo-adjuvant chemotherapy have greatly advanced, only 25-60% of the osteosarcoma patients respond well, and the rest of the patients with advanced or metastatic osteosarcoma would eventually die due to resistance to anticancer treatments [2]. It is indicated that OS cells originate from several sources, including the undifferentiated mesenchymal stem cells (MSCs), which could develop into cancer stem cells (CSCs) after accumulation of genetic and epigenetic alterations [3]. CSCs play a key role in the onset, treatment resistance, tumor metastasis, and relapse by utilizing their self-renewal and differentiation properties [4]. For example, the embryonal transcriptional regulator Oct-4, SOX2, and Nanog are previously found in osteosarcoma tissues and cells and play important roles in its development [5]. Besides, activation of Wnt/β-catenin pathway leads to transition of osteosarcoma cells into a stem-like state, and subsequently causes chemotherapy failure [6]. Hence, suppressing the cancer cell stemness is a potential therapeutic approach for osteosarcoma. Of note, a recent study determined that MAFB is differentially expressed between tumor section and normal tissues in osteosarcoma, and MAFB transcriptionally regulated the portion of CSCs in osteosarcoma [7].

Levobupivacaine is a commonly used local anesthetics in clinical application [8]. Recent studies disclosed that administration of local anesthetics could also affect the development of cancers in various cancers through inhibiting cell growth and migration [9, 10]. For instance, it has been indicated that levobupivacaine treatment caused impeded growth and metastasis of melanoma cells [11]. Jose and colleagues revealed that levobupivacaine treatment led to a combined suppression of glycolysis and oxidative phosphorylation in human prostate cancer cells [12]. Nevertheless, whether levobupivacaine affects the function of CSCs, especially in osteosarcoma, is not clear.

Lysine acetyltransferase 5 (KAT5) belongs to acetylases family that epigenetically regulate gene expression, and is the first human acetylases identified to be capable of inducing acetylation of both non-histone and histone proteins [13, 14]. KAT5 is one of the most important lysine acetyltransferases (KATs), which has recently been suggested as a potential therapeutic target for cancers, such as breast and colon cancers [13, 15, 16]. Meanwhile, KAT5 plays a crucial role in the modulation of osteosarcoma progression [17, 18].

In this study, we exploited the function of local anesthetics levobupivacaine on osteosarcoma, identified suppressed proliferation, migration and stemness of osteosarcoma cells after treatment. Additionally, levobupivacaine administration inhibited the expression of KAT5, which epigenetically regulated the acetylation of MAFB and subsequently cancer cell stemness. Therefore, levobupivacaine might act as a novel therapeutic agent for osteosarcoma via targeting CSCs.

## RESULTS

### Levobupivacaine inhibits osteosarcoma cell proliferation *in vitro* and *in vivo*

**Figure 1:**
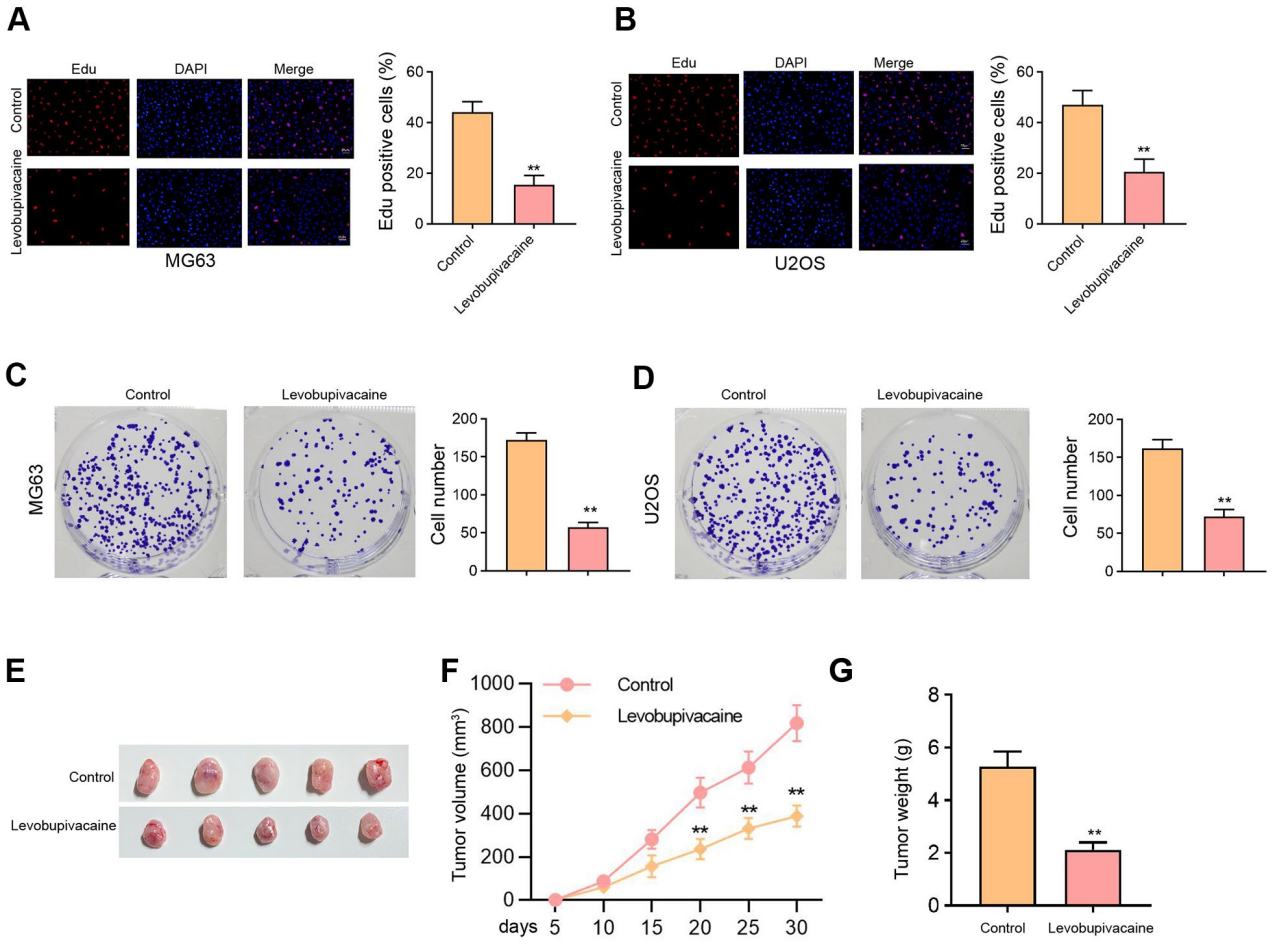
**Levobupivacaine inhibits osteosarcoma cell proliferation *in vitro* and *in vivo*.** (**A**–**D**) MG63 and U2OS cells were treated with levobupivacaine (2 mM). (**A**, **B**) Cell proliferation was detected by Edu assays. (**C**, **D**) Cell proliferation was measured by colony formation assays. (**E**–**G**) The nude mice (n = 5) were injected with MG63 cells and 40 μmol/kg levobupivacaine. The tumor xenograft was conducted in the mice. (**E**) The tumor images, (**F**) the tumor volume, (**G**) and the tumor weight were shown. mean ± SD, ** *P* < 0.01.

We initially assessed the effect of levobupivacaine on osteosarcoma cell proliferation. Our data showed that the treatment of levobupivacaine suppressed numbers of Edu-positive MG63 and U2OS cells (Figure 1A, 1B). Meanwhile, the colony numbers of MG63 and U2OS cells were repressed by levobupivacaine (Figure 1C, 1D). The tumor xenograft analysis showed that levobupivacaine significantly repressed the MG63 cell growth in the nude mice (Figure 1E–1G).

### Levobupivacaine induces osteosarcoma cell apoptosis and represses invasion

**Figure 2:**
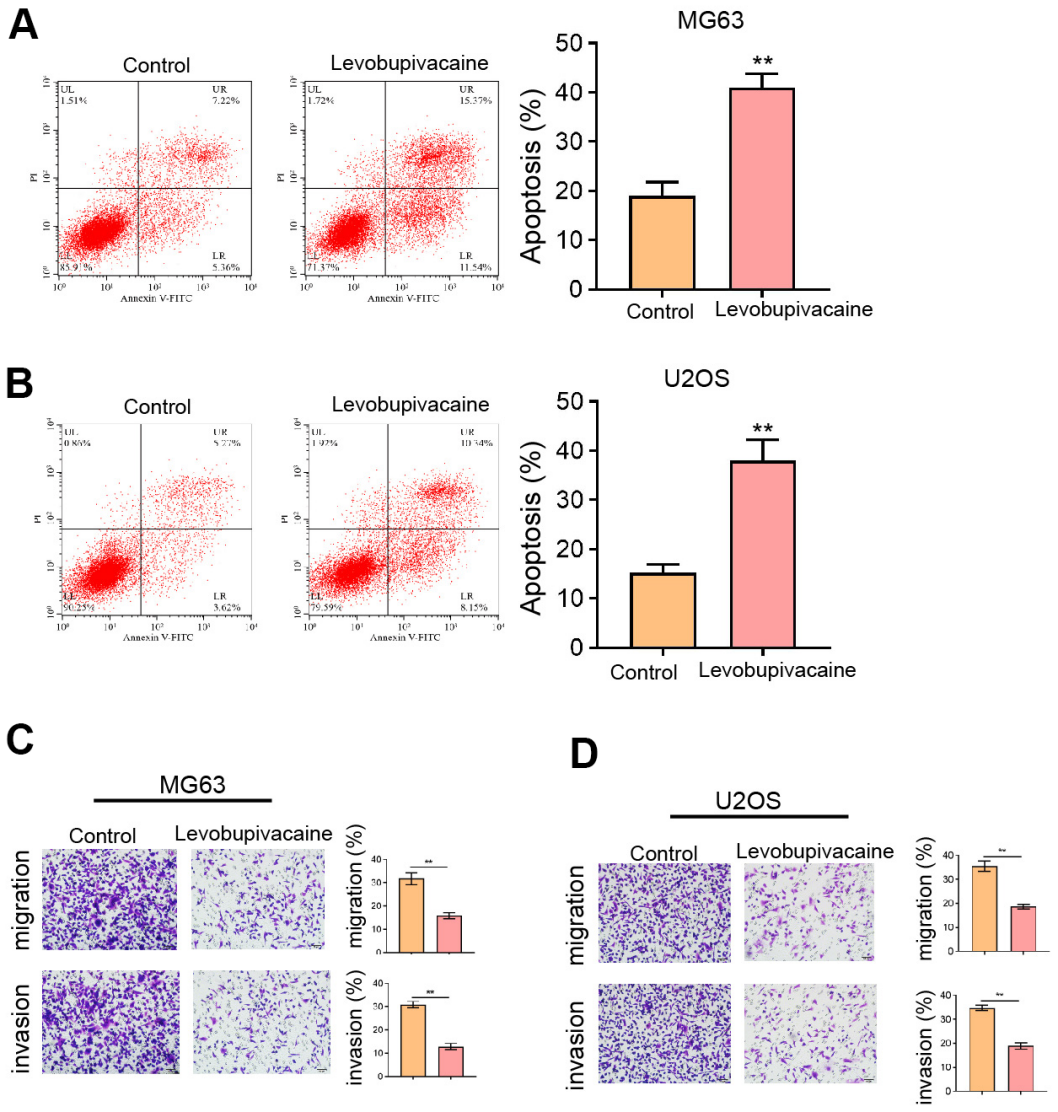
**Levobupivacaine induces osteosarcoma cell apoptosis and represses invasion.** (**A**–**D**) MG63 and U2OS cells were treated with levobupivacaine (2 mM). (**A,** **B**) Cell apoptosis was analyzed by Annexin V/PI apoptosis detection kit. (**C,** **D**) Cell invasion and migration were detected by transwell assays. mean ± SD, ** *P* < 0.01.

Moreover, we found that the treatment of levobupivacaine improved the apoptosis rate of MG63 and U2OS cells (Figure 2A, 2B). The invasion and migration abilities of MG63 and U2OS cells were attenuated by levobupivacaine as well (Figure 2C, 2D).

### Levobupivacaine suppresses CSCs properties of osteosarcoma cells

**Figure 3:**
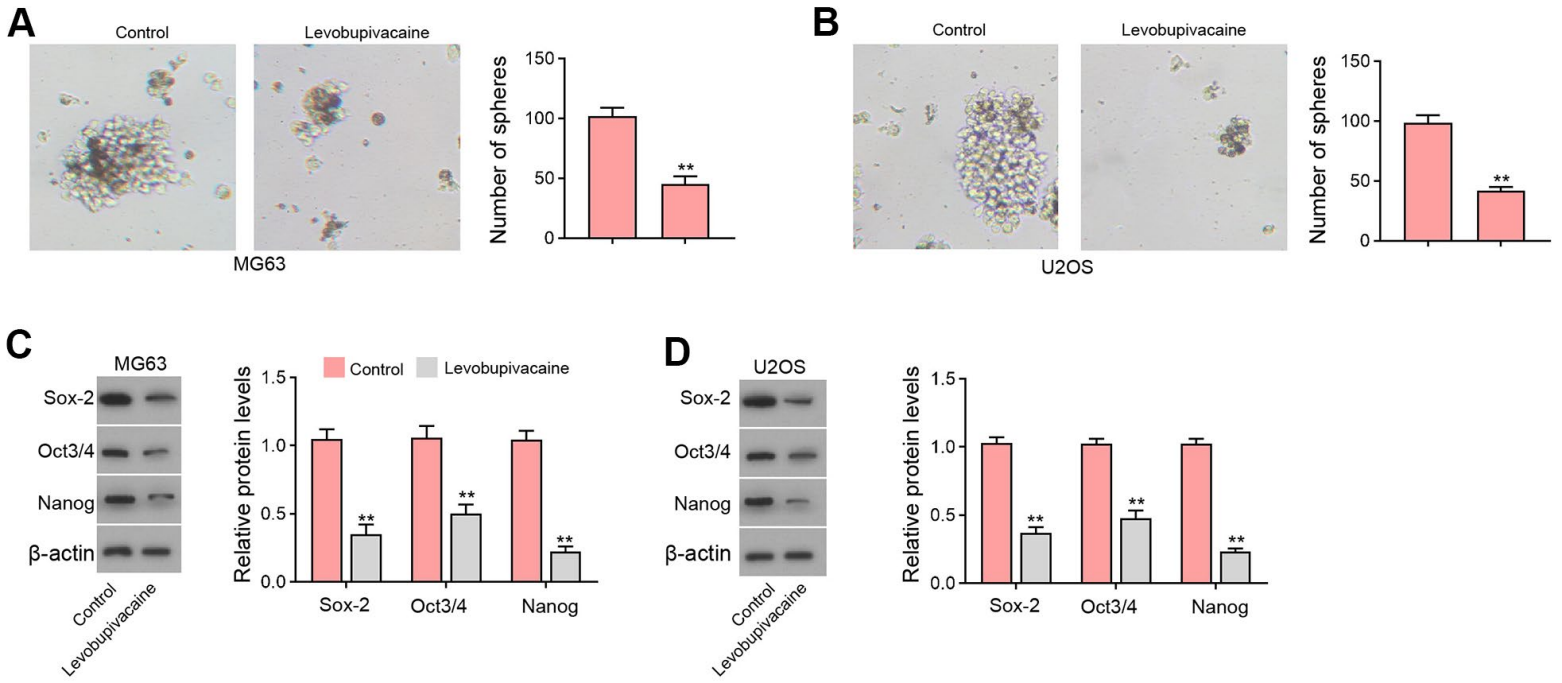
**Levobupivacaine suppresses CSCs properties of osteosarcoma cells.** (**A**–**D**) MG63 and U2OS cells were treated with levobupivacaine (2 mM). (**A,** **B**) The stemness was measured by sphere formation assays. (**C,** **D**) The protein levels of Sox-2, Oct3/4, and Nanog were analyzed by Western blot. mean ± SD, ** *P* < 0.01.

Furthermore, we observed that the sphere formation capabilities of MG63 and U2OS cells were repressed by levobupivacaine (Figure 3A, 3B). The protein levels of Sox-2, Oct3/4, and Nanog were inhibited by the treatment of levobupivacaine in MG63 and U2OS cells (Figure 3C, 3D).

### Levobupivacaine epigenetically inhibits MAFB expression by reducing KAT5 in osteosarcoma cells

**Figure 4:**
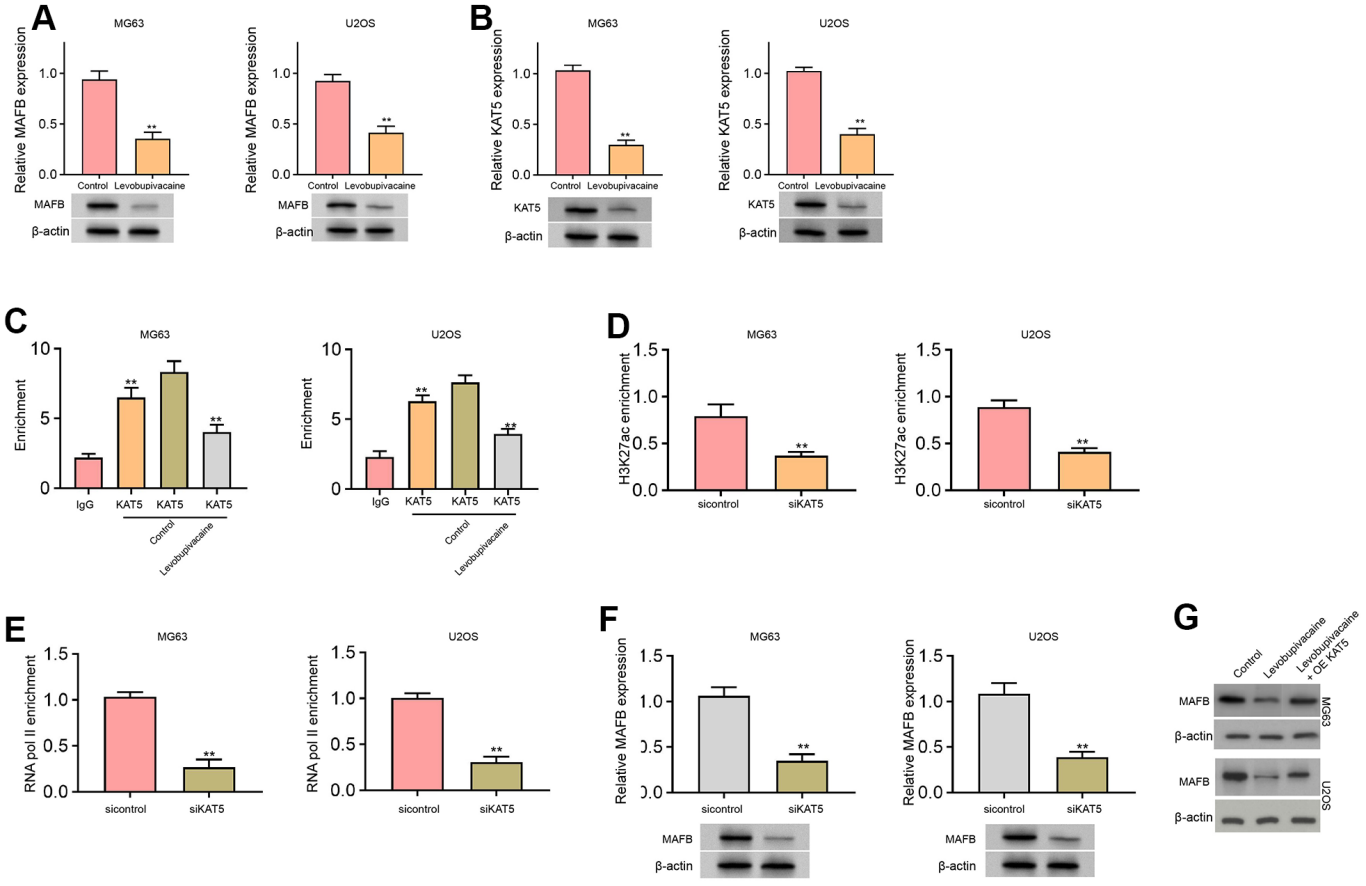
**Levobupivacaine epigenetically inhibits MAFB expression by reducing KAT5 in osteosarcoma cells.** (**A**–**C**) MG63 and U2OS cells were treated with levobupivacaine (2 mM). The expression of MAFB and KAT5 was detected by qPCR and Western blot analysis. (**C**) The enrichment of KAT5 on MAFB promoter was analyzed by ChIP analysis. (**D**–**F**) MG63 and U2OS cells were transfected with KAT5 siRNA. (**D**) The enrichment of H3K27ac on MAFB promoter was determined by ChIP analysis. (**E**) The enrichment of RNA polymerase II on MAFB promoter was tested by ChIP analysis. (**F**) MAFB expression was assessed by qPCR and Western blot analysis. (**G**) MAFB expression was examined by Western bot in MG63 and U2OS cells treated with levobupivacaine, or co-treated with levobupivacaine and KAT5 overexpression vector. mean ± SD, ** *P* < 0.01.

Next, we identified that the treatment of levobupivacaine inhibited MAFB and KAT5 expression in MG63 and U2OS cells (Figure 4A, 4B). We observed that KAT5 could enriched in the promoter region of MAFB in MG63 and U2OS cells, while levobupivacaine treatment could repressed the enrichment (Figure 4C). The effectiveness of KAT5 depletion by siRNA was validated in the cells (Supplementary Figure 1A, 1B). Meanwhile, the suppression of KAT5 by siRNA repressed the enrichment of histone H3 acetylation at lysine 27 (H3K27ac) on promoter of MAFB (Figure 4D). Consistently, the enrichment of RNA polymerase II on promoter of MAFB was reduced by the depletion of KAT5 in MG63 and U2OS cells (Figure 4E). The expression of MAFB was decreased by KAT5 knockdown in MG63 and U2OS cells (Figure 4F). Moreover, the expression of MAFB was repressed by levobupivacaine, while the overexpression of KAT5 could reverse the repression of MAFB in MG63 and U2OS cells (Figure 4G). Moreover, we validated that the expression of MAFB and KAT5 was enhanced in clinical osteosarcoma tissues and the expression of MAFB was positively correlated with KAT5 in the tissues (Supplementary Figure 2).

### The inhibition of KAT5 reduces cell proliferation and induces cell apoptosis in osteosarcoma cells

**Figure 5:**
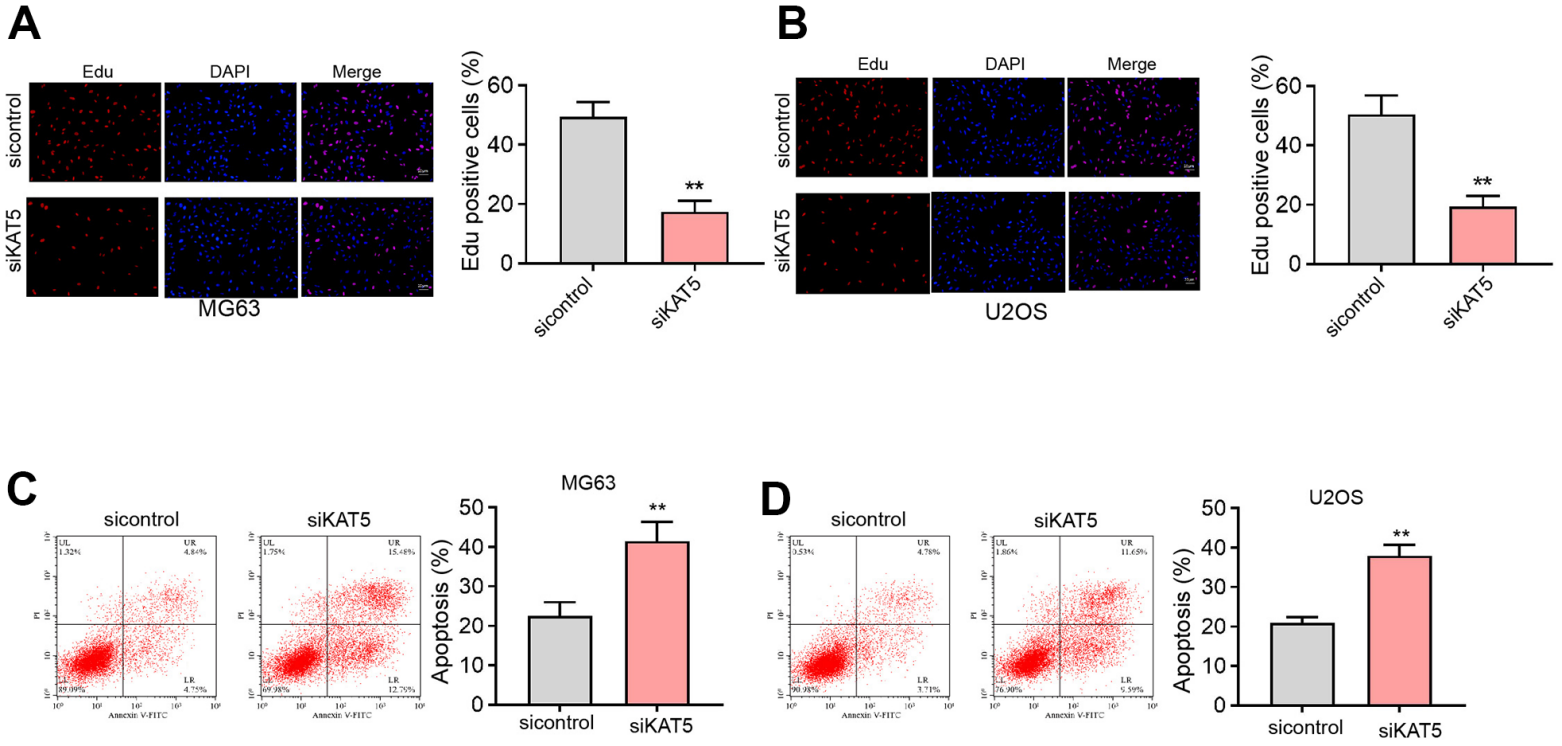
**The inhibition of KAT5 reduces cell proliferation and induces cell apoptosis in osteosarcoma cells.** (**A**–**D**) MG63 and U2OS cells were transfected with KAT5 siRNA. (**A**, **B**) Cell proliferation was detected by Edu assays. (**C**, **D**) Cell apoptosis was analyzed by Annexin V/PI apoptosis detection kit. mean ± SD, ** *P* < 0.01.

We then identified that the suppression of KAT5 by siRNA was able to attenuate the counts of Edu-positive MG63 and U2OS cells (Figure 5A, 5B). Meanwhile, the cell apoptosis of MG63 and U2OS cells was induced by the depletion of KAT5 as well (Figure 5C, 5D).

### The inhibition of KAT5 represses CSCs properties of osteosarcoma cells

**Figure 6:**
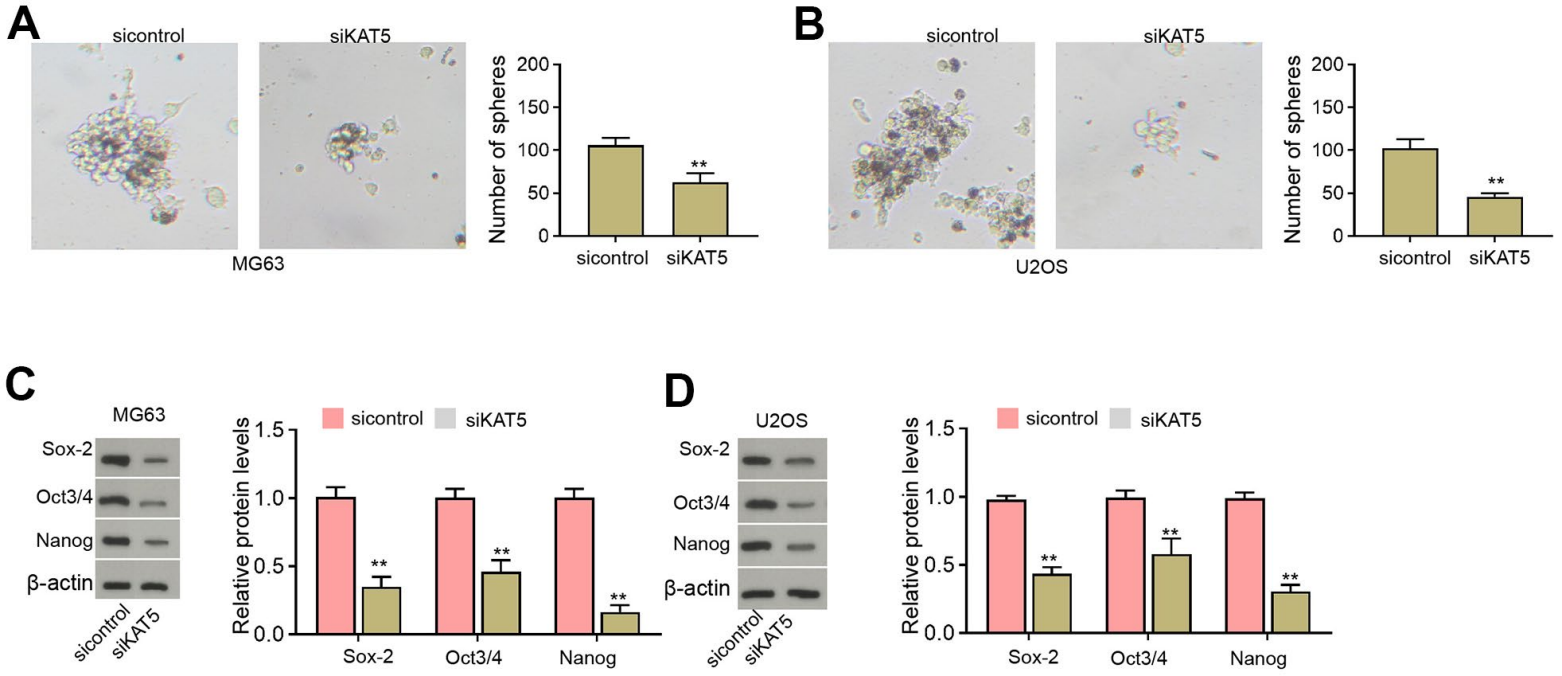
**The inhibition of KAT5 represses CSCs properties of osteosarcoma cells.** (**A**–**D**) MG63 and U2OS cells were transfected with KAT5 siRNA. (**A**, **B**) The stemness was measured by sphere formation assays. (**C**, **D**) The protein levels of Sox-2, Oct3/4, and Nanog were analyzed by Western blot. mean ± SD, ** *P* < 0.01.

Moreover, we found that the sphere formation abilities of MG63 and U2OS cells were inhibited by the depletion of KAT5 (Figure 6A, 6B). The protein levels of Sox-2, Oct3/4, and Nanog were repressed by the inhibition of KAT5 in MG63 and U2OS cells (Figure 6C, 6D).

### Levobupivacaine attenuates cell proliferation and CSCs properties of osteosarcoma cells by regulating KAT5/MAFB

**Figure 7:**
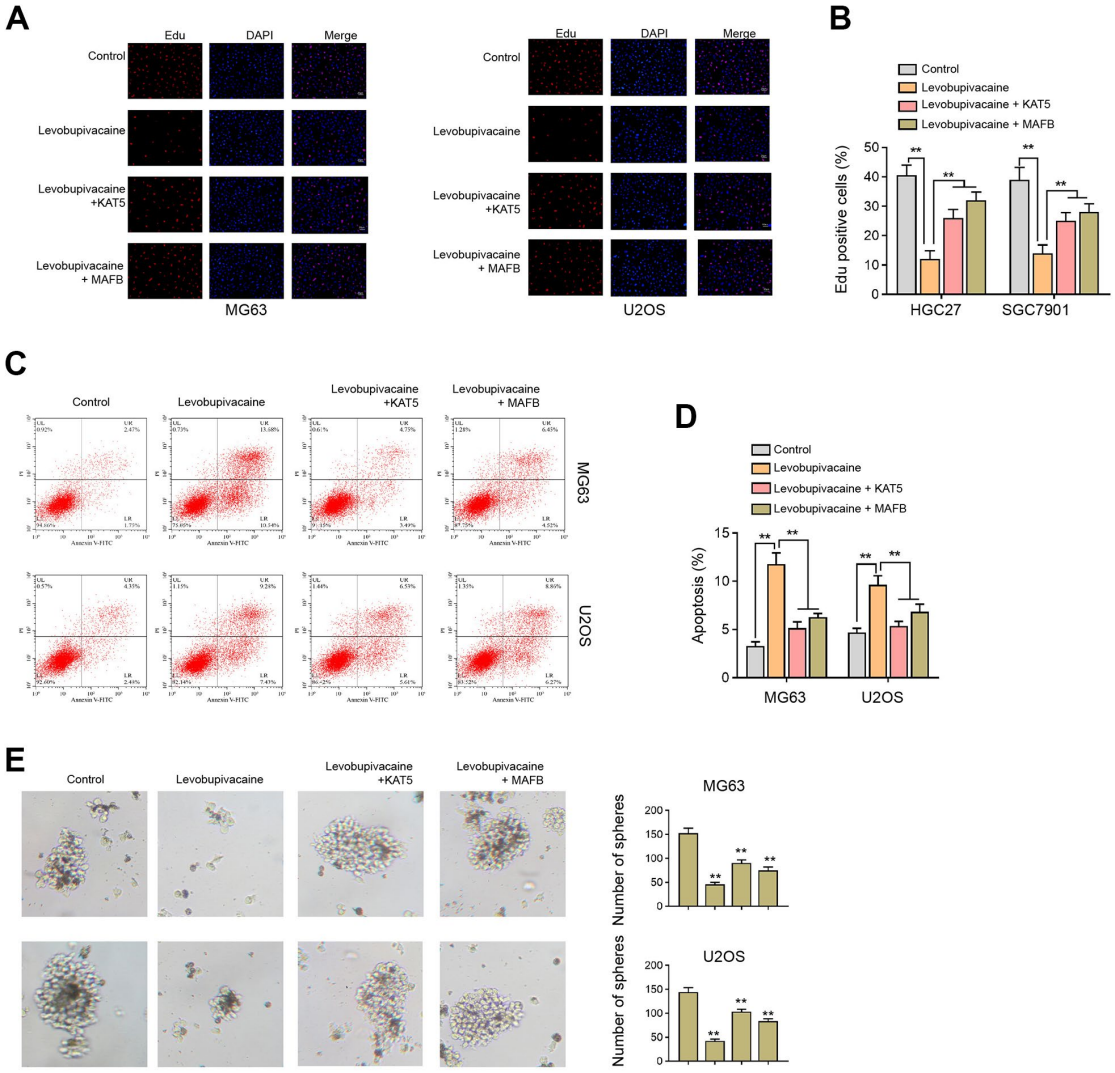
**Levobupivacaine attenuates cell proliferation and CSCs properties of osteosarcoma cells by regulating KAT5/MAFB.** (**A**–**E**) MG63 and U2OS cells were treated with levobupivacaine, or co-treated with levobupivacaine and KAT5 or MAFB overexpression vectors. (**A,** **B**) Cell proliferation was detected by Edu assays. (**C**, **D**) Cell apoptosis was analyzed by Annexin V/PI apoptosis detection kit. (**E**) The stemness was measured by sphere formation assays. mean ± SD, ** *P* < 0.01.

The effectiveness of KAT5 overexpression was validated in the cells (Supplementary Figure 1B). Next, our data demonstrated that the Edu-positive MG63 and U2OS cells were decreased by the treatment of levobupivacaine and the overexpression of KAT5 or MAFB could rescued the phenotype (Figure 7A, 7B). Levobupivacaine enhanced apoptosis rate of MG63 and U2OS cells, in which KAT5 or MAFB overexpression reversed apoptosis (Figure 7C, 7D). The sphere formation abilities inhibited by levobupivacaine could be rescued by the overexpression of KAT5 or MAFB (Figure 7E).

## DISCUSSION

Osteosarcoma serves as a prevalent bone cancer and CSCs present a critical role in the onset, treatment resistance, tumor metastasis by their self-renewal and differentiation properties. Levobupivacaine is a widely applied local anesthetics in the clinic and has been reported anti-cancer activities. However, the function of levobupivacaine in osteosarcoma and CSCs is still obscure. In the presented investigation, we discovered a novel function of levobupivacaine in repressing CSCs in osteosarcoma.

Previous studies have shown the anti-cancer activities of levobupivacaine. It has been reported that levobupivacaine represses proliferation and enhances apoptosis by targeting the PI3K/Akt pathway in breast cancer [19]. Levobupivacaine reduces cell growth of colon cancer [20]. Levobupivacaine inhibits migration and proliferation in melanoma and triple-negative breast cancer [11]. Several anti-tumor agents have been reported to inhibited CSCs in osteosarcoma. Wogonin represses stemness of osteosarcoma cells by targeting ROS-related signaling [21]. Resveratrol inhibits CSCs of osteosarcoma by the inhibition of STAT3 signaling [22]. Dioscin attenuates tumor growth and CSCs of osteosarcoma by Akt/GSK3 pathway [23]. Bruceine D decreases CSCs and tumor growth by targeting STAT3 signaling in osteosarcoma [24]. In this study, we found that the treatment of levobupivacaine inhibited proliferation of osteosarcoma cells *in vitro*. The tumor xenograft analysis demonstrated that levobupivacaine significantly suppressed the osteosarcoma cell growth in the nude mice. The treatment of levobupivacaine enhanced the apoptosis rate and attenuated invasion and migration of osteosarcoma cells. The stemness properties of osteosarcoma cells were repressed by levobupivacaine. These data present a new experimental basic of the novel role of levobupivacaine in suppressing osteosarcoma cell proliferation and stemness, indicating the potential of the therapeutic application of levobupivacaine in the treatment of osteosarcoma. The doses and clinical exercise of levobupivacaine in osteosarcoma need to explore in future clinical research.

Moreover, it has been reported that KAT5 regulates the acetylation of KDM2B to enhance progression of osteosarcoma [18]. Circular RNA circRHOT1enhances non-small cell lung cancer progression by epigenetically enhancing C-MYC expression through recruiting KAT5/H3K27ac axis [25]. Ketamine inhibits proliferation and promotes ferroptosis and apoptosis of breast cancer cells via targeting KAT5 KAT5/H3K27a/GPX4 axis [26]. MAFB plays a crucial function in regulating CSCs properties. The deficiency of p53 promotes progression of myeloma by reprogramming hematopoietic stem cells to malignant plasma cells by regulating MAFB [27]. MAFB contributes to tumorigenesis and stemness of osteosarcoma by targeting Sox9 [7]. But the correlation of MAFB with KAT5 and H3K27ac is still unreported. Our mechanical study showed that levobupivacaine inhibited MAFB and KAT5 expression in osteosarcoma cells. KAT5 epigenetically enhanced MAFB expression in osteosarcoma cells. Levobupivacaine repressed MAFB expression by reducing KAT5. Our finding identifies a new epigenetic mechanism of levobupivacaine-mediated cancer development. Meanwhile, we provide a new function of KAT5 for the epigenetically inducing MAFB expression in the regulation of CSCs properties of osteosarcoma. The clinical significance and association of KAT5 and MAFB need more evidence and investigations.

In summary, we concluded that local anesthetic levobupivacaine inhibited stemness of osteosarcoma cells by epigenetically repressing MAFB though reducing KAT5 expression.

## MATERIALS AND METHODS

### Cell culture and transfection

The osteosarcoma cell lines MG63 and U2OS were ordered from the Shanghai Institute of Biochemistry and Cell Biology (China), and cultured in MEM medium (HyClone, USA) and MoCoy’s 5a medium (HyClone), respectively. All medium were added with 10% fetal bovine serum (FBS, Gibco) and 1% penicillin/streptomycin solution (Thermo, USA). All cells were placed at a 37° C humidified incubator containing 5% CO_2_. Overexpressing plasmids inserted with the CDS regions of KAT5 (pCMV-KAT5) and MAFB (pCMV-MAFB), and small interfering RNA targeting KAT5 (siKAT5) and the corresponding negative control were obtained from GenePharma (China). For cell transfection, the plasmids or siRNAs were mixed in transfection reagent lipofectamine 2000 (Invitrogen, USA) in opti-MEM medium (HyClone) and added into cultured MG63 and U2OS for 24 hours. Cells were then collected for subsequent experiments. The dose of levobupivacaine was used at 2 mM according to the previous report [28].

### Osteosarcoma samples

The primary chondroma and osteosarcoma tissues were collected from 27 patients before the neo-adjuvant chemotherapy. The patients were histologically characterized by a pathologist using the criteria described by the World Health Organization. The application of the samples was underwritten informed consent of the patients, and all study protocols were approved by the Ethics Committee of The First Hospital of Lanzhou University.

### Cell growth

Colony formation experiment was conducted to evaluate cell proliferation. The MG63 and U2OS cells were seeded in 6-well plates (600 cells/well) and treated with Levobupivacaine at 0 or 2mM for 14 days till the visible colonies formed. The colonies were sained by 0.5% crystal violet in methanol solution for 15 minutes, photographed by a microscope (Leica, Germany) and counted.

For 5-ethynyl-2′-deoxyuridine (EdU) assay, MG63 and U2OS cells were placed in 96-well plates and treated by levobupivacaine or indicated transfection. The cells were labelled by EdU and stained by Hoechst 33342 in accordance with the protocol of EdU-labeling kit (Thermo, USA). The intensity of positive staining was captured by a fluorescence microscope (Leica) and counted.

### Apoptosis

MG63 and U2OS cells were placed in 96-well plates and treated by levobupivacaine or indicated transfection for 24 hours. Then the cells were collected and stained by a Annexin V/PI apoptosis detection kit (Thermo) under manufacturer’s instruction. In short, cells were suspended in binding buffer and stained by FITC-conjugated Annexin V reagent and PI for 15 minutes in dark, respectively. The labelled cells were detected by a flow cytometer (BD Biosciences, USA).

### Transwell assay

Transwell assays analyzed the invasion and migration by using a Transwell plate (Corning, USA) according to the manufacturer's instruction. To analyze the cell migration, the cells were cultured for 24 hours and resuspended by serum-free culture medium, then plated into the apical chamber of transwell at a density of 5 × 10^3^ cells/well. The culture medium was made up to 150 μL and the basolateral chamber was added with 600 μL complete culture medium. After 24 hours, culture at 37° C and 5% CO_2_, the cells were fixed through 4% paraformaldehyde 10 minutes, stained by crystal violet dye for 20 minutes, followed by the analysis using the intelligent biological navigator (Olympus, Tokyo, Japan). The migrated cells were recorded and calculated by using the ImageJ software.

To analyze the cell invasion, Matrigel was melted overnight at 4° C and diluted by presold serum-free culture medium (ratio 8: 1). The medium (50 μL) was plated into the transwell polycarbonate membrane with a pore diameter of 8 μm, making all the wells were covered by Matrigel at 37° C for 2 hours. The cells were cultured for 24 hours and resuspended by serum-free culture medium, then plated into the apical chamber of transwell at 1 × 10^5^ cells/well, the medium was made up to 150 μL. The basolateral chamber was added with 600μL complete medium with 50% FBS. After 24 hours, the cells were fixed using 4% paraformaldehyde for 15 minutes, stained by crystal violet dye for 10 minutes. The invaded cells were analyzed and calculated by using the ImageJ software.

### Western blotting

Total protein was extracted from MG63 and U2OS cells by a Protein Extraction Reagent (Thermo). An equivalent amount of 30 μg cell lysates were loaded and separated in the 10% SDS-PAGE and shifted to the nitrocellulose membrane (Bio-Rad, USA). Membranes were soaked in 5% bovine serum albumin (BSA) for 1 hour, followed by incubation with primary antibodies against MAFB (ab243902), Sox-2 (ab92494), Oct3/4 (ab230429), Nanog (ab109250), GAPDH (ab8245) and subsequently with corresponding secondary antibodies. All antibodies were purchased from Abcam (USA) and diluted according to the manufacturer’s protocols. The bands were visualized by an enhanced chemiluminescent reagent (ECL, Millipore, USA) in a gel image system (Bio-Rad, USA).

### Sphere formation assay

MG63 and U2OS were subjected to appropriate transfection, and seeded in ultra-low attachment 24-well plates (Corning) at a density of 200 cells per well. The culture medium consists of DMEM/F12 medium (Hyclone) added with EGF, bFGF, methyl cellulose, and B27 supplement (PeproTech, USA) following manufacturer’s protocols. Cells were cultured for two weeks and the medium was changed every three days. The formed spheres were photographed by a microscope (Leica).

### Real-time PCR

Total RNA was isolated from MG63 and U2OS cells via a TRIzol reagent (Sigma) after indicated treatment in each experiment. The extracted RNA was subjected to Maxima H Minus cDNA Synthesis Master Mix (Thermo) to obtain cDNAs, and subsequent quantification by an Applied Biosystem SYBR kit (Thermo). The relative expression of MAFB and KAT5 were normalized to the internal control GAPDH. Primers were listed:

MAFB, sense, 5’-GACGCAGCTCATTCAGCAG-3’, antisense, 5’-CTCGCACTTGACCTTGTAGGC-3’;

KAT5, sense, 5’-AACAAACGTCTGGATGAATGGG-3’, antisense, 5’-AGGAAGTCCGTTCTTAGTGGG-3’;

GAPDH, sense, 5’- sense, 5’-ACAACTTTGGTATCGTGGAAGG3’, antisense, 5’-GCCATCACGCCACAGTTTC-3’.

### Chromatin immunoprecipitation (ChIP) assay

ChIP assays were conducted to determine the epigenetic regulatory of MAFB by using a ChIP assay kit (Thermo) following the manufacturer’s description. In short, MG63 and U2OS cells were transfected with siKAT5 or NC, collected, fixed with formaldehyde and lysed. The extracted DNA were sonicated to short fragments of around 200 to 500 bp. Subsequently, the fragments were incubated with antibody against KAT5, H3K27Ac, or RNA Polymerase II (Abcam) at 4° C for 12 hours in rotation, and the following binding with Protein A agarose beads (Thermo) for 3 hours at room temperature. The precipitants were washed and the DNA-protein complex was striped off the beads. PCR assay was used to measure the level of DNA segments.

### *In vivo* tumorigenicity

SCID/Nude mice at 4-weeks old were subcutaneously injected with osteosarcoma cells (10^6^ cells/site) suspended in Matrigel and DMEM medium (1:1), and were fed with free access to water and food. Levobupivacaine were administrated at a dose of 40 μmol/kg every day [28]. The size of the subcutaneous tumors was measured every 3 days and calculated by the following formula: volume = 0.5 × length × width^2^. Mice were ordered from Vital River Laboratory Animal Technology (China). All animal experiments were approved by the Institutional Animal Care and Use Committee (IACUC) of the First Hospital of Lanzhou University.

### Statistical analysis

Each experiment was conducted at least three times independently and data was presented as mean ± SD. Statistical differences were analyzed using Student’s t-test or one-way ANOVA by using the SPSS 17.0 software (USA). A p value less than 0.05 was regarded as statistically significant.

## Supplementary Material

Supplementary Figures
