# Supplementary material for: Local anesthetic levobupivacaine inhibits stemness of osteosarcoma cells by epigenetically repressing MAFB though reducing KAT5 expression
Source: Aging (Albany NY). 2022 Mar 25;14(6):2793–804. doi: 10.18632/aging.203975 (PMC9004559; doi:10.18632/aging.203975)
Supplement: Supplementary Figures [file aging-14-203975-s001.pdf]

SUPPLEMENTARY FIGURES

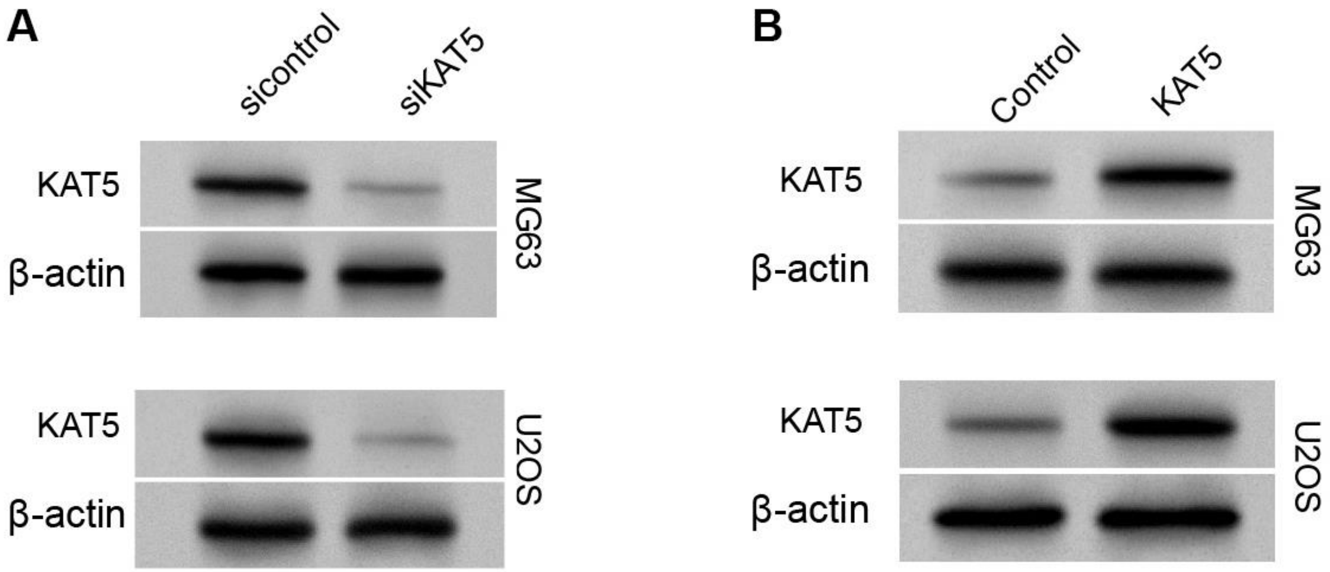

Supplementary Figure 1. The validation of expression KAT5. (**A, B**) The expression of KAT5 was measured by Western blot analysis in MG63 and U2OS cells treated as the indication.

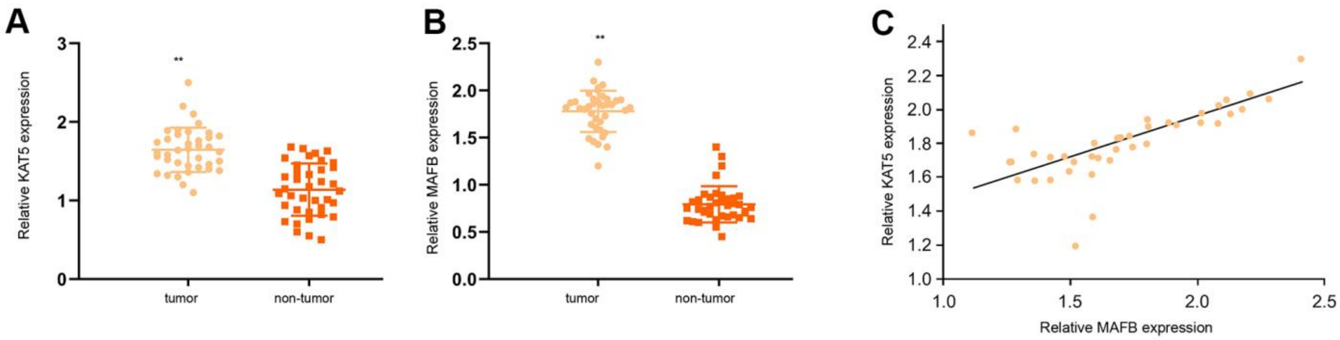

Supplementary Figure 2. The expression of KAT5 and MAFB is enhanced in clinical osteosarcoma samples. (**A**–**C**) The expression and correlation of MAFB and KAT5 was measured by qPCR in clinical osteosarcoma tissues (n=27). ** $P < 0.01$.
